# *QuickStats:* Rates* of Injury^†^ from Sports, Recreation, and Leisure Activities^§^ Among Children and Adolescents Aged 1–17 Years, by Age Group — National Health Interview Survey,^¶^ United States, 2015–2017

**DOI:** 10.15585/mmwr.mm6820a6

**Published:** 2019-05-24

**Figure:**
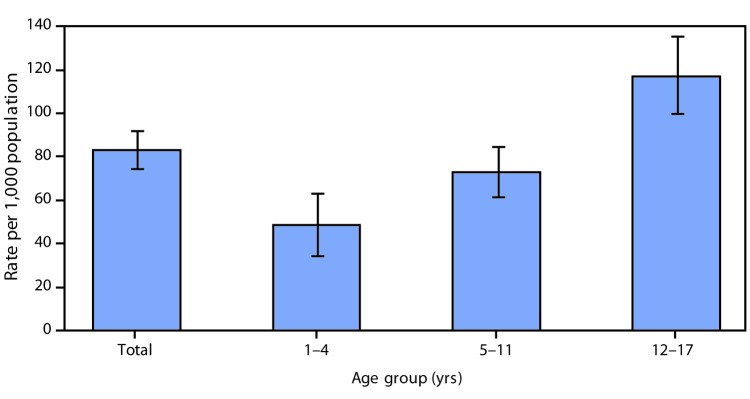
In 2015–2017, the rate of sports, recreation, and leisure injuries among children and adolescents aged 1–17 years was 82.9 per 1,000 population. The rate of sports, recreation, and leisure injuries increased with age from 48.4 for those aged 1–4 years, to 72.7 for those aged 5–11 years, and to 117.1 for those aged 12–17 years.

**Source:** National Health Interview Survey, 2015–2017.

For more information on this topic, CDC suggests the following link: https://www.cdc.gov/safechild/index.html.

